# Innovative fusion models: elevating preoperative gross ETE prediction in thyroid cancer patients

**DOI:** 10.1038/s41598-026-43230-3

**Published:** 2026-03-11

**Authors:** Ting Pan, Fan Wu, Juanjuan Cai, Yu Zhang, Zhiyu Xing

**Affiliations:** 1 Cancer Center, Department of Pathology, Zhejiang Provincial People’s Hospital (Affiliated People’s Hospital), Hangzhou Medical College, Hangzhou, 310014 Zhejiang China; 2 Department of Oncological Surgery, Affiliated Hangzhou First People’s Hospital, Westlake University School of Medicine, Hangzhou, 310006 Zhejiang China; 3 Department of Ultrasound, Affiliated Hangzhou First People’s Hospital, School of Medicine, Westlake University School of Medicine, Hangzhou, 310006 Zhejiang China

**Keywords:** Papillary thyroid carcinoma, Peritumoral, Radiomics, Extrathyroidal extension, Cancer imaging, Thyroid cancer

## Abstract

The intratumoral and peritumoral architectural heterogeneities of papillary thyroid carcinoma (PTC) are important in preoperative prediction of gross extrathyroidal extension (Gross ETE). This study systematically evaluated and compared the predictive efficacies of deep learning, radiomics, and their combined approach (Deep Learning-Radiomics, DLR) in predicting Gross ETE in PTC patients using ultrasound imaging. This retrospective study from three hospitals, between 01/01/2018, and 12/31/2022, included 4,542 PTC patients, divided into training (*n* = 3,179) and testing (*n* = 1,363) sets in a 7:3 ratio. Preoperative ultrasound images and clinical data were collected to develop radiomics and deep learning models based on different tumor expansion regions (5/10/15/20 pixels). A nomogram prediction model was developed by integrating multi-regional radiomics features and key clinical parameters. Model performance was assessed using metrics such as the area under the curve (AUC), sensitivity, and specificity. Feature importance was evaluated using SHapley Additive exPlanations (SHAP) analysis, and model interpretability was analyzed with Gradient-weighted Class Activation Mapping (Grad-CAM). In the test cohort, the radiomics model with 15 pixel expansion (AUC: 0.796) and the ResNet101 deep learning model (AUC: 0.832) showed optimal performance. The DLR model incorporating 15 pixel peritumoral features (DLRexpand15) combined with clinical parameters achieved superior predictive performance (AUC: 0.849, accuracy: 0.857, and specificity: 0.888). SHAP analysis identified deep learning features as the primary predictors in the fusion model, while Grad-CAM visualization confirmed spatial concordances between model-activated regions and histopathological invasion patterns. The DLRexpand15-based nomogram integrating clinical indicators provided an effective tool for preoperative prediction of Gross ETE in PTC patients. Strategic incorporation of peritumoral information significantly enhanced the predictive capacity of both radiomics and deep learning models. This multimodal approach provided clinically useful insights for surgical planning and risk stratification.

**Supplementary Information:**

The online version contains supplementary material available at 10.1038/s41598-026-43230-3.

## Introduction

Accurate preoperative staging of papillary thyroid carcinoma (PTC) is crucial for guiding treatment and assessing prognosis. Gross extrathyroidal extension (Gross ETE), a key marker of tumor aggressiveness, significantly impacts surgical planning and long-term patient management^1^. Under the American Joint Committee on Cancer eighth edition staging system, Gross ETE is closely linked to higher tumor recurrence risks and lower survival rates^2^. However, traditional ultrasound assessment, which depends on subjective morphological features (such as a nodule-capsule contact area of > 25% and deformation of adjacent structures), has limitations in sensitivity (40%−65%) and inter-observer consistency, leading to a higher risk of preoperative misjudgment^3,4^.

Recent advances in radiomics have revolutionized tumor heterogeneity analysis through high-throughput extraction of quantitative imaging biomarkers. Imaging-driven machine learning (ML) has emerged as a transformative decision-support tool in oncology^5–8^. Contemporary ML implementations follow two distinct paradigms: (1) radiomics pipelines combining handcrafted feature engineering with shallow learning architectures (e.g., support vector machines and decision trees), and (2) deep learning (DL) approaches using convolutional neural networks to autonomously extract discriminative patterns from raw image data^9^. Notably, existing investigations predominantly focus on intratumoral characteristics, while underutilizing peritumoral microenvironmental signatures, including textural alterations in capsular regions and infiltration patterns in adjacent tissues^4^. Emerging evidence suggests that dynamic tumor-host interface interactions correlate strongly with local invasive behavior, implying that synergistic analysis of intratumoral and peritumoral radiomics features may enhance gross ETE prediction accuracy^10^, thereby emphasizing the clinical imperative for spatial heterogeneity integration.

The nomogram, a visual predictive tool that quantifies multifactorial risks and facilitates individualized clinical decision-making, has demonstrated significant utility in oncological prognosis assessments^11^. This study therefore integrated intratumoral and peritumoral ultrasound radiomics features with clinical parameters to develop a nomogram for predicting gross ETE. Using multicenter retrospective cohort validation, we addressed three critical questions: (1) whether peritumoral imaging features independently enhanced gross ETE prediction efficacy, (2) whether the multiparametric nomogram outperformed conventional single indicators and empirical diagnostics, and (3) whether the model maintained generalization capability across imaging devices and institutions. The results provided a novel method for preoperative precision assessment of gross ETE, and also expanded the applications of radiomics in the management of thyroid carcinomas.

## Methods

### Study cohort and clinical data

**Fig. 1:**
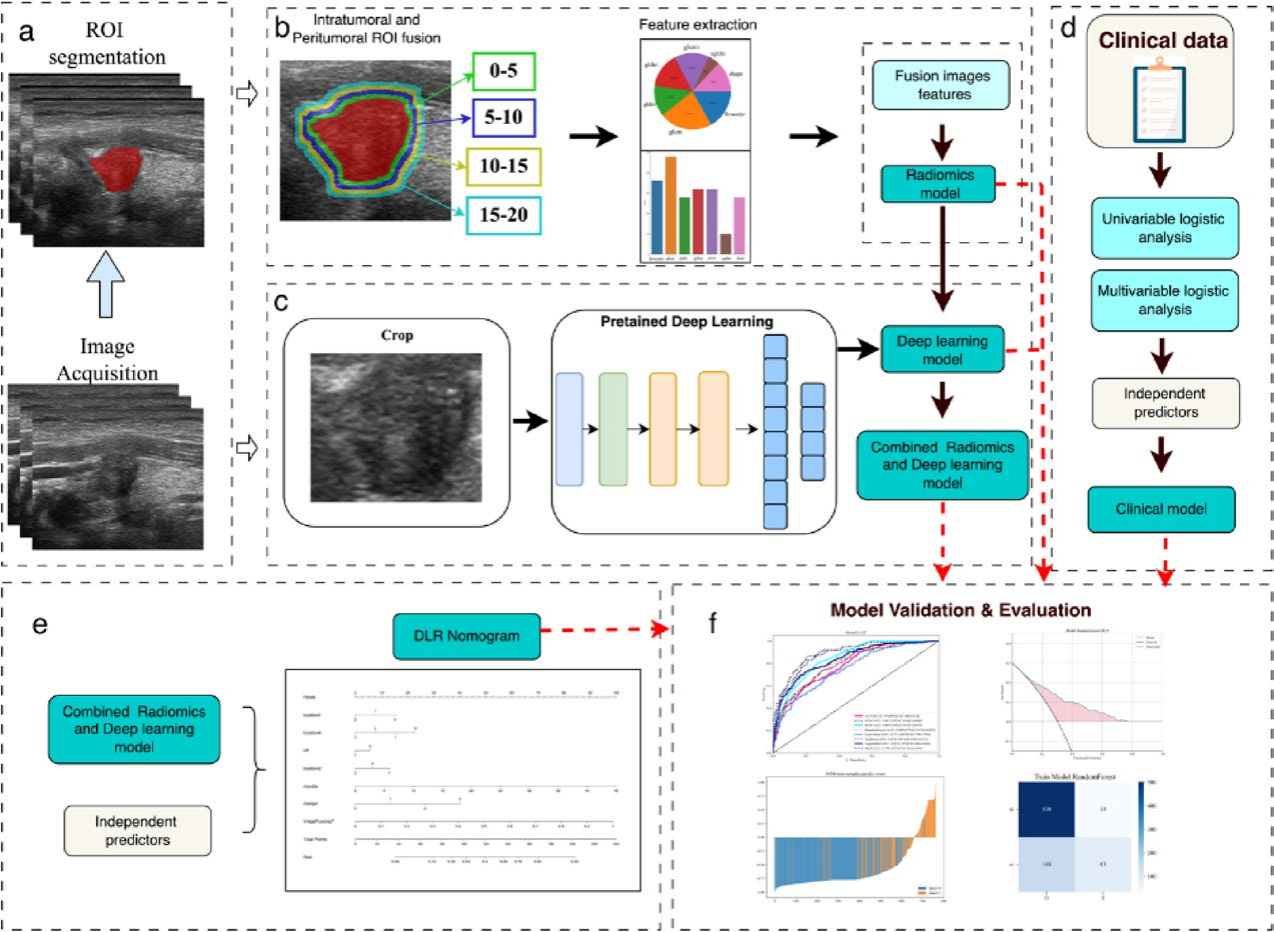
Workflow scheme of the deep learning and radiomics analysis.

The study was approved by the Ethics Committee of Hangzhou First People’s Hospital (2025ZN054-1). The dataset comprised PTC patients who underwent preoperative ultrasound (US) examinations between 01/01/2018 and 12/31/2022 at two imaging centers: Affiliated Hangzhou First People’s Hospital, School Of Medicine, Westlake University (Hangzhou, Zhejiang, China) and Yuhuangding Hospital, Affiliated to Qingdao University (Yantai, Shandong, China), followed by surgical confirmation. Inclusion criteria required the following: (a) postoperative pathological confirmation of PTC, (b) available preoperative ultrasound images, and (c) complete surgical pathology records. Exclusion criteria included the following: (a) incomplete/poor quality US images, (b) history of concurrent malignancies, (c) rare histological variants, or (d) missing clinical data (Fig. 1).

The training and test cohorts were randomly stratified from Centers A (*n* = 4,482) and B (*n* = 60). Two board certified radiologists with 5 years of thyroid imaging experience independently reviewed all US images, excluding studies with insufficient qualities, artifacts, or diagnostic disagreements. The final consensus dataset included 4,542 patients. A 7:3 ratio allocated samples into training (*n* = 3,179) and testing (*n* = 1,363) cohorts (Supplementary Method S1). This study was conducted according to the principles of the Declaration of Helsinki. Approval was granted by the Ethics Committee of Hangzhou First People’s Hospital (2025ZN054-1).

To better predict Gross ETE in PTC, this study adapted the ACR TI-RADS (2017) and ATA Guidelines (2015) in its specific criteria. Gross ETE was defined as gross tumor invasion identified at the time of surgery and confirmed by histopathologic review^11^. The study meticulously collected clinical and ultrasound features of patients, including the following ultrasound features: echogenicity; anechoic; hyperechoic; hypoechoic; marked hypoechoic, aspect ratio > 1 (taller-than-wide) < 1 (wider-than-tall); calcification; absent/large comet-tail artifacts; macrocalcification; peripheral/rim calcification, and punctate echogenic foci.

### Ultrasound image acquisition

The diagnostic equipment used for thyroid examination were the Mylab 70 XVG and Mylab color Doppler ultrasound systems, with a probe frequency of 7 − 13 MHz. Patients were placed in a supine position before surgery to fully expose the neck area. Once a suspicious malignant nodule was identified, physicians interpreted and documented the two-dimensional ultrasound features of the nodule. Two experienced ultrasound physicians evaluated preoperative tumor information using ultrasound, without knowing the pathological results of all thyroid nodules.

### Image acquisition

#### ROI segmentation

In this study, the Region of Interest (ROI) delineation process was conducted under a rigorous three-stage quality control protocol. Initially, two board-certified radiologists specializing in medical imaging independently performed manual annotations using ITK-SNAP software (version 3.8.0). These manually curated segmentation masks subsequently served as the training dataset for the development of an automated segmentation model.

For automatic ROI identification, this study used the DeepLabv3 architecture^12^. The model leveraged atrous convolution strategies within a modified ResNet backbone and incorporated an Atrous Spatial Pyramid Pooling module, enabling effective capture of multi-scale contextual information while preserving spatial resolution. The initial segmentation results generated by this deep learning model were then systematically reviewed and when necessary, manually refined by the radiologists to ensure precision. To resolve any inter-observer discrepancies, a consensus-building mechanism was implemented, wherein discordant segmentations were adjudicated by a senior neuroradiologist with over 25 years of clinical experience. This multi-tiered annotation strategy ultimately yielded the final gold-standard segmentation masks. Critically, all radiologists involved in the manual annotation and refinement processes were blinded to the pathological diagnoses of the patients.

####  Peritumoral region dilation

The peritumoral zone surrounding the tumor boundary was systematically analyzed to assess its impact on predictive modeling. Using the automated mask expansion tool in the OnekeyAI platform, we created concentric expansions around the tumor boundaries at 5-pixel radial intervals. This methodological approach generated multiple peritumoral rings (Fig. 1) to quantitatively evaluate how varying distances from the tumor edge affected model performance metrics. The expansion process maintained anatomical consistency through automated spatial calibration, preserving tissue structure relationships during region enlargement.

### Radiomics procedure

#### Feature extraction

In our study, the handcrafted features were divided into three categories: (I) geometry, (II) intensity, and (III) texture. Geometric characteristics focused on the three-dimensional morphological aspects of the tumor, illustrating its structural configuration in space. The intensity descriptors quantified the first-order statistical attributes of voxel intensities within the tumor region, revealing the tumor’s internal uniformity or heterogeneity. In addition, the texture descriptors analyzed the intricate patterns by examining the second-order and higher-order spatial arrangements of intensities, thereby capturing the complex spatial interrelations between voxels. These texture descriptors were obtained through various methods, including gray-level co-occurrence matrix, gray-level run length matrix, gray-level size zone matrix, and neighborhood gray-tone difference matrix. All handcrafted features were extracted using an in-house developed feature analysis program powered by Pyradiomics (http://pyradiomics.readthedocs.io).

#### Feature selection

We applied Z-score normalization to all extracted features and used a *t*-test to evaluate their statistical significance, selecting only those with a p-value < 0.05. To address collinearity, we analyzed the correlations between features using Pearson’s correlation coefficient and excluded one feature from any pair where the correlation coefficient exceeded 0.9. Subsequently, we used Lasso regression within a 10-fold cross-validation framework to determine the optimal regularization parameter, λ. This process effectively streamlined the feature set, retaining only the most predictive and informative features.

#### Radiomics signature

Using this LASSO-driven feature selection, we developed a radiomics risk model using machine-learning algorithms, including Logistic Regression (LR), NaiveBayes, Support Vector Machine (SVM), ExtraTrees, and AdaBoost. Comparative analyses were conducted to evaluate each model’s performance. We also determined the advantages of integrating multi-modal features through feature fusion, discovering how combining multiple imaging modalities boosted predictive accuracy.

#### Peri-radiomics signature

We used the same rigorous feature selection process as that used for the Intra Radiomics Signature. The final model was built using the same set of machine learning algorithms. This ensured consistency and comparability in our approach to both intra- and peri-tumoral analyses.

### Deep learning

#### Feature extraction

We used pretrained models on real images, including ResNet18, ResNet34, ResNet50, ResNet101, ResNet152, DenseNet121, DenseNet161, DenseNet169, DenseNet201, Inception_v3, and Manasnet0_5, to extract DL features from each standardized ROI image. The model was trained using the stochastic gradient descent optimizer with an initial learning rate of 0.01, decayed by the cosine annealing algorithm for 50 epochs, and a batch size of 32. We used the fixed network parameters after model training as feature extractors. To enhance model generalizability and mitigate overfitting, we applied random flipping for data augmentation.

#### Model construction

Using the trained deep learning model, we extracted 2,048 dimensional deep features from the penultimate average pooling layer. To enhance generalization and reduce overfitting, we applied principal component analysis to reduce the feature dimension to 512, followed by Z-score normalization for feature scaling. During feature selection, we combined Spearman’s correlation analysis with LASSO regression. Finally, using the selected deep learning features, we constructed the prediction model by employing multiple traditional machine learning classifiers.

### Construction of the fusion model

To enhance the AUC, this study developed a DLR model combining DL and radiomics features. We concatenated DL features (*n* = 512) extracted from the penultimate average pooling layer of pretrained deep learning models with radiomics features (*n* = 1,561), forming combined DLR features (*n* = 2,077) for each image. Multiple models were trained with varying parameters and evaluated using a test cohort via five-fold cross-validation. LASSO regression was used to select the most relevant features for identifying gross ETE. The selected features were then input into classifiers to generate predictions. To determine the optimal classifier, five classifiers (LR, NaiveBayes, SVM, ExtraTrees, and AdaBoost) were developed and assessed on a development dataset (70% training and 30% validation). The implementation steps were the same as those used in the radiomics feature processing.

### Metrics

We rigorously assessed the diagnostic efficacy of our deep learning model in the test cohort by constructing Receiver Operating Characteristic (ROC) curves to evaluate its discriminative ability. Model calibration was analyzed using calibration curves and further validated using the Hosmer-Lemeshow goodness-of-fit test. Additionally, we used Decision Curve Analysis (DCA) to evaluate the clinical utility of our predictive models, helping to clarify their potential benefits in practical clinical settings.

### Visualization and auxiliary diagnosis function of the DL model

To assist radiologists in diagnoses, the model’s diagnostic results were presented in a visually interpretable manner. Our DL model automatically localized the nodule within the US image and delineated its boundaries using a mask, thereby revealing the nodule’s location and shape. To further enhance the interpretability of the DL model for humans, we used the Grad-CAM technique, which effectively highlighted the model’s focus areas.

### Interpretability of machine learning (ML) models

After identifying the optimal radiomics, DL, and fusion models, we used the SHAP method to further analyze feature importance, to identify the most impactful variables. Features were ordered based on the magnitude of their SHAP values, enabling us to identify the key predictive factors within our patient cohort.

### Statistical analysis

The normality of clinical features was verified using the Shapiro-Wilk test. Continuous variables were analyzed with *t*-tests or Mann-Whitney U tests, depending on their distribution. Categorical variables were assessed using Chi-square (χ²) tests. Table 1 details the baseline characteristics of all cohorts. Cohorts with p-values > 0.05 showed no significant difference, confirming an unbiased grouping.

**Table 1:** Baseline characters of our cohorts.

Feature_Name	ALL	Train(*N* = 3179)	Test(*N* = 1363)	*p*
Age	45.14 ± 12.35	45.15 ± 12.28	45.12 ± 12.52	0.953
Maximum diameter	9.26 ± 7.21	9.26 ± 7.25	9.27 ± 7.12	0.766
Sex				0.517
Female	3469(76.38)	2419(76.09)	1050(77.04)	
Male	1073(23.62)	760(23.91)	313(22.96)	
Hashimoto’s thyroiditis				0.0493
No	3444(75.83)	2384(74.99)	1060(77.77)	
Yes	1098(24.17)	795(25.01)	303(22.23)	
Echo				0.970
Hyperechoic	188(4.14)	133(4.18)	55(4.04)	
Hypoechoic	4219(92.89)	2952(92.86)	1267(92.96)	
Markedly hypoechoic	135(2.97)	94(2.96)	41(3.01)	
Aspect ratio				0.631
< 1	1665(36.66)	1173(36.90)	492(36.10)	
≥ 1	2877(63.34)	2006(63.10)	871(63.90)	
Marigin				0.929
	495(10.90)	352(11.07)	143(10.49)	
Well-defined	1854(40.82)	1290(40.58)	564(41.38)	
III-defined	2116(46.59)	1483(46.65)	633(46.44)	
lobulated	77(1.70)	54(1.70)	23(1.69)	
Calcification				0.986
None	1616(35.58)	1131(35.58)	485(35.58)	
Macrocalcification	1165(25.65)	817(25.70)	348(25.53)	
Peripheral calcification	189(4.16)	130(4.09)	59(4.33)	
Microcalcification	1572(34.61)	1101(34.63)	471(34.56)	

All data analyses used the OnekeyAI platform, version 4.9.1, with Python 3.7.12. Statistical evaluations used Statsmodels 0.13.2. Radiomics feature extraction used PyRadiomics 3.0.1, machine learning through Scikit-learn 1.0.2, and deep learning frameworks were built on PyTorch 1.11.0, enhanced by CUDA 11.3.1 and cuDNN 8.2.1.

## Results

### Clinical features of patient characteristics

A total of 4,542 patients were obtained from two centers. Patients were excluded for the following reasons: US examination not performed or unsatisfactory US image quality (*n* = 328), previous history of other malignant tumors (*n* = 128), incomplete clinicopathological information (*n* = 182), incomplete surgical information (*n* = 18), and nonclassic PTC or specific histologic subtypes (*n* = 62). The final training dataset included 3,179 patients (mean age: 45.15 ± 12.28 years), and the testing dataset included 1,363 patients (mean age: 45.12 ± 12.52 years), with a total of 4,542 ultrasound images. No significant difference was observed between the datasets (*p* = 0.01) (Table 1). Inclusion and exclusion criteria are provided in Supplemental Figure S1.

### Univariate and multivariate analyses

In our study, we performed a comprehensive univariate analysis of all clinical features, placing particular emphasis on the evaluation of odds ratios (OR) and their corresponding p-values for each variable. This ratio played a pivotal role in the development of our final fusion model. In our experimental process, significant features identified through univariable screening were incorporated into the construction of the Clinical Signature.

Univariate analysis showed that echo, maximum diameter, margin, age, sex, aspect ratio, Hashimoto’s thyroiditis, and calcification were associated with gross ETE in the training dataset (Table 2). Variables with p-values < 0.05 in the univariate analysis were further examined in a multivariate model. The results indicated that echo, maximum diameter, and margin remained independent predictors of the gross ETE.

**Table 2:** Univariable and multivariable analysis of clinical features.

Feature_Name	Univariate OR	Univariate 95%CI	*P* value	Multivariate OR	Multivariate 95%CI	*P* value
Echo	0.372	0.356–0.389	< 0.05	0.259	0.212–0.315	< 0.05
Maximum Diameter	0.884	0.876–0.891	< 0.05	1.078	1.067–1.089	< 0.05
marigin	0.313	0.295–0.333	< 0.05	1.223	1.067–1.401	0.015
Age	0.959	0.957–0.961	< 0.05	0.998	0.991–1.004	0.510
Sex	0.148	0.124–0.177	< 0.05	0.857	0.687–1.071	0.256
Aspect ratio	0.131	0.117–0.147	< 0.05	0.839	0.694–1.014	0.128
Hashimoto’s thyroiditis	0.136	0.113–0.163	< 0.05	0.934	0.753–1.158	0.600
Calcification	0.436	0.414–0.46	< 0.05	0.928	0.863–0.997	0.087

### Radiomics, deep learning, and DLR analysis

In radiomics analysis, 1,561 features were extracted from each ROI. Following Pearson’s correlation analysis and least absolute shrinkage and selection operator (LASSO) regression, 19, 25, 29, and 39 features were retained from 5-, 10-, 15-, and 20-pixel expanded regions (denoted as expand 5/10/15/20), respectively, to construct radiomics signatures. The detailed visualizations of the LASSO process—including the cross‑validation curve, coefficient trajectories, and histogram of selected feature weights—are provided in Supplementary Figure S2. Table 3 compares the predictive capabilities of intratumoral versus progressively expanded peritumoral radiomics features derived from ultrasound imaging, with bold entries indicating models exhibiting optimal AUC values and minimal overfitting. Comprehensive evaluations showed the superior predictive performance of expanded peritumoral regions over tumor-centric analysis, with the 15-pixel expansion achieving maximal discriminative power. Consequently, expand15 radiomics features were selected for subsequent modeling.

**Table 3:** Gross ETE prediction performance of tumoral and peritumoral regions in ultrasound.

Dilation Distance	Group	ACC	AUC	95% CI	SENS	SPEC	PPV	NPV
0 mm	train	0.73	0.776	0.751–0.802	0.703	0.733	0.258	0.949
	test	0.663	0.748	0.708–0.787	0.746	0.651	0.246	0.944
5 mm	train	0.801	0.801	0.776–0.825	0.687	0.816	0.341	0.95
	test	0.762	0.79	0.753–0.827	0.673	0.774	0.291	0.945
10 mm	train	0.78	0.8	0.775–0.825	0.712	0.789	0.318	0.952
	test	0.76	0.788	0.751–0.824	0.679	0.771	0.29	0.946
15 mm	train	**0.813**	**0.812**	**0.788–0.835**	0.676	0.832	0.357	0.949
	test	**0.781**	**0.796**	**0.759–0.834**	0.685	0.794	0.314	0.948
20 mm	train	0.773	0.813	0.789–0.836	0.718	0.781	0.311	0.952
	test	0.808	0.787	0.749–0.825	0.636	0.831	0.342	0.943

*ACC* accuracy; *AUC* area under the curve; *CI* confidence interval; *SENS* sensitivity; *SPEC* specificity; *PPV* Positive Predictive Value; *NPV* Negative Predictive Value.

For deep learning implementation, 16 distinct architectures were evaluated (Supplementary Table S1). The predictive model utilizing features extracted by ResNet101 demonstrated superior performance in both training and test cohorts, outperforming comparative models. The ResNet101-based model showed excellent calibration consistency and optimal clinical utility on DCA (Fig. 3).

**Fig. 2:**
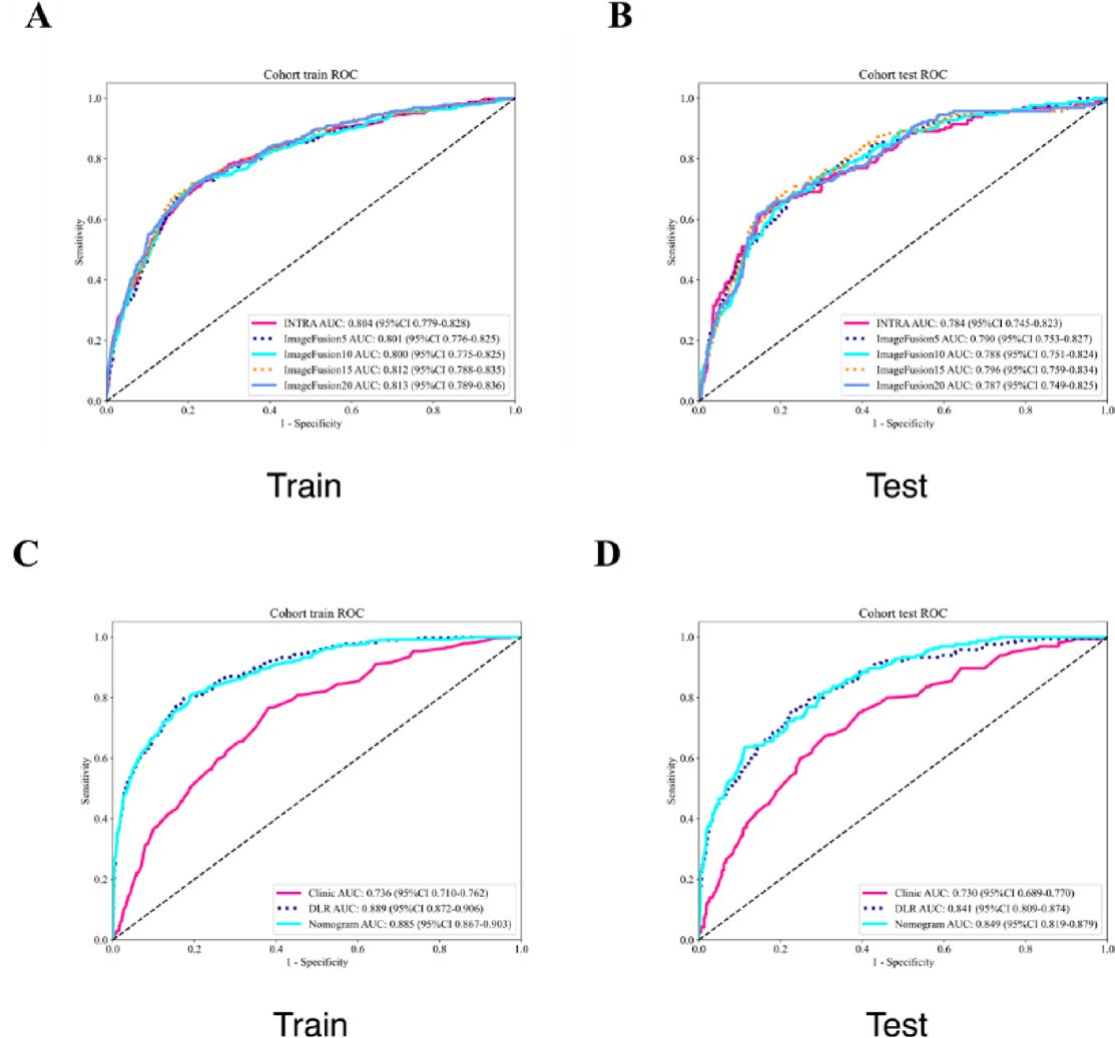
Receiver operating characteristic (ROC) curves of the three models.

**Fig. 3:**
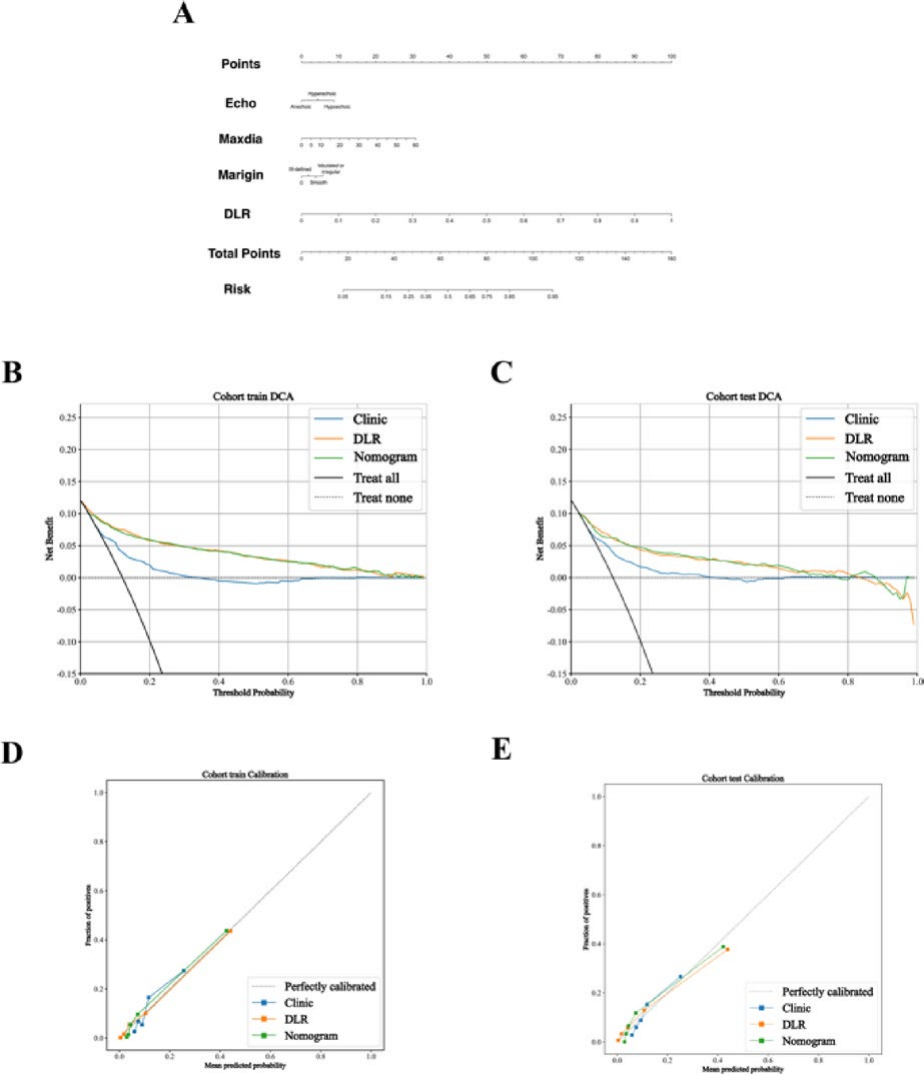
A nomogram for predicting gross extrathyroidal extension (ETE) and its performance evaluation. **A** The nomogram integrates the combined radiomics signature and key clinical factors, (e.g., tumor location, Hashimoto’s thyroiditis, maximum diameter, and margin) to calculate the individual probability of the gross ETE. Points are assigned for each variable, summed, and projected to the “Total Points” axis to derive the corresponding “ETE Probability.”, **B**, **C** Decision curve analysis for the nomogram in the, (B) training and, (C) test cohorts. The y-axis represents the net benefit. The “Nomogram” curve, (red line) shows superior clinical utilities across most probability thresholds, when compared to the “Treat All” and “Treat None” strategies., **D**, **E** Calibration curves of the nomogram in the, **D** training and, (E) test cohorts. The x-axis represents the nomogram-predicted probability of gross ETE, and the y-axis represents the observed frequency of gross ETE. The solid blue line closer to the ideal diagonal dashed line indicates good agreement between predictions and observations.

The final DLRexpand15 model synergistically integrated expand15 radiomics features with ResNet101 deep learning outputs. Compared to stand-alone radiomics or DL models, DLRexpand15 achieved significant performance gains (Fig. 2; Table 4). Specific metrics included: training cohort: AUC: 0.901 (95% CI: 0.873 − 0.929), accuracy: 0.830, sensitivity: 0.834, specificity: 0.830; test cohort: AUC: 0.842 (95% CI: 0.815 − 0.869), accuracy: 0.757, sensitivity: 0.739, and specificity: 0.760 (Fig. 3).

**Table 4:** Metrics on different signatures.

Cohort	Signature	ACC	AUC	95%CI	SENS	SPEC	PPV	NPV
Train	ImageFusion15mm	0.822	0.889	0.872–0.906	0.798	0.825	0.386	0.967
Train	DL	0.844	0.926	0.913–0.938	0.845	0.844	0.428	0.975
Train	DLR	0.830	0.901	0.885–0.918	0.834	0.830	0.404	0.973
Test	ImageFusion15mm	0.737	0.841	0.809–0.874	0.794	0.729	0.287	0.963
Test	DL	0.804	0.832	0.799–0.865	0.691	0.820	0.345	0.951
Test	DLR	0.757	0.842	0.811–0.874	0.739	0.760	0.298	0.955

*ACC* accuracy, *AUC* area under curve, *CI* confidence interval, *SENS* sensitivity, *SPEC* specificity, *PPV* Positive Predictive Value, *NPV* Negative Predictive Value.

Probability histograms (Supplementary Figure S3) revealed enhanced interclass discriminations in DLRexpand15, with distinct probability distribution peaks for positive/negative cases. Confusion matrix analysis (Supplementary Figure S4) confirmed reduced false positives, while maintaining high true positive rates, validating the model’s clinical reliability.

### Performance evaluation of prediction models

Our investigation further enhanced clinical applicability by integrating clinical variables with the fusion model into a comprehensive nomogram. This integrated model showed improved predictive performance with the following metrics: training cohort: AUC: 0.885 (95% CI: 0.854 − 0.916), accuracy: 0.808, sensitivity: 0.808, specificity: 0.808; test cohort: AUC: 0.849 (95% CI: 0.817 − 0.881), and accuracy: 0.857, sensitivity: 0.630, and specificity: 0.888. Additional diagnostic parameters, including positive and negative predictive values, are detailed in Table 5. Decision curve analysis showed superior net benefit across probability thresholds for the integrated model, when compared with clinical-only benchmarks, demonstrating enhanced clinical utility for surgical decision making (Fig. 3B-C). Calibration analysis using the Hosmer-Lemeshow test confirmed good agreement between predicted probabilities and observed outcomes in the test cohort (Fig. 3D-E).

**Table 5:** Metrics of different signatures.

Cohort	Signature	ACC	AUC	95%CI	SENS	SPEC	PPV	NPV
Train	Clinic	0.638	0.736	0.7104–0.7618	0.762	0.621	0.217	0.95
Train	ImageFusion15mm	0.822	0.889	0.8723–0.9062	0.798	0.825	0.386	0.967
Train	Nomogram	0.808	0.885	0.8674–0.9029	0.808	0.808	0.367	0.968
Test	Clinic	0.711	0.73	0.6892–0.7704	0.63	0.722	0.238	0.934
Test	ImageFusion15mm	0.737	0.841	0.8089–0.8738	0.794	0.729	0.287	0.963
Test	Nomogram	0.857	0.849	0.8192–0.8791	0.630	0.888	0.437	0.946

*ACC* accuracy, *AUC* area under curve, *CI* confidence interval, *SENS* sensitivity, *SPEC* specificity, *PPV* Positive Predictive Value, *NPV* Negative Predictive Value.

The DeLong test was used across both training and testing cohorts to assess inter-model performance differences. Our combined nomogram showed statistically significant superiority over clinical models, however, its incremental improvement versus the DLR model was less substantial, suggesting diminishing marginal utility from clinical-DLR integration (Supplementary Figure S5). Integrated Discrimination Improvement, quantifying enhanced risk stratification through predicted probability distribution comparisons, showed significant discriminative gains for the nomogram, relative to baseline models. Net reclassification improvement analysis, evaluating accuracy improvements in case reclassifications, showed superior categorical recalibrations, with 68% of misclassified cases being correctly restratified (Supplementary Figure S5).

### Model interpretation and visualization

**Fig. 4:**
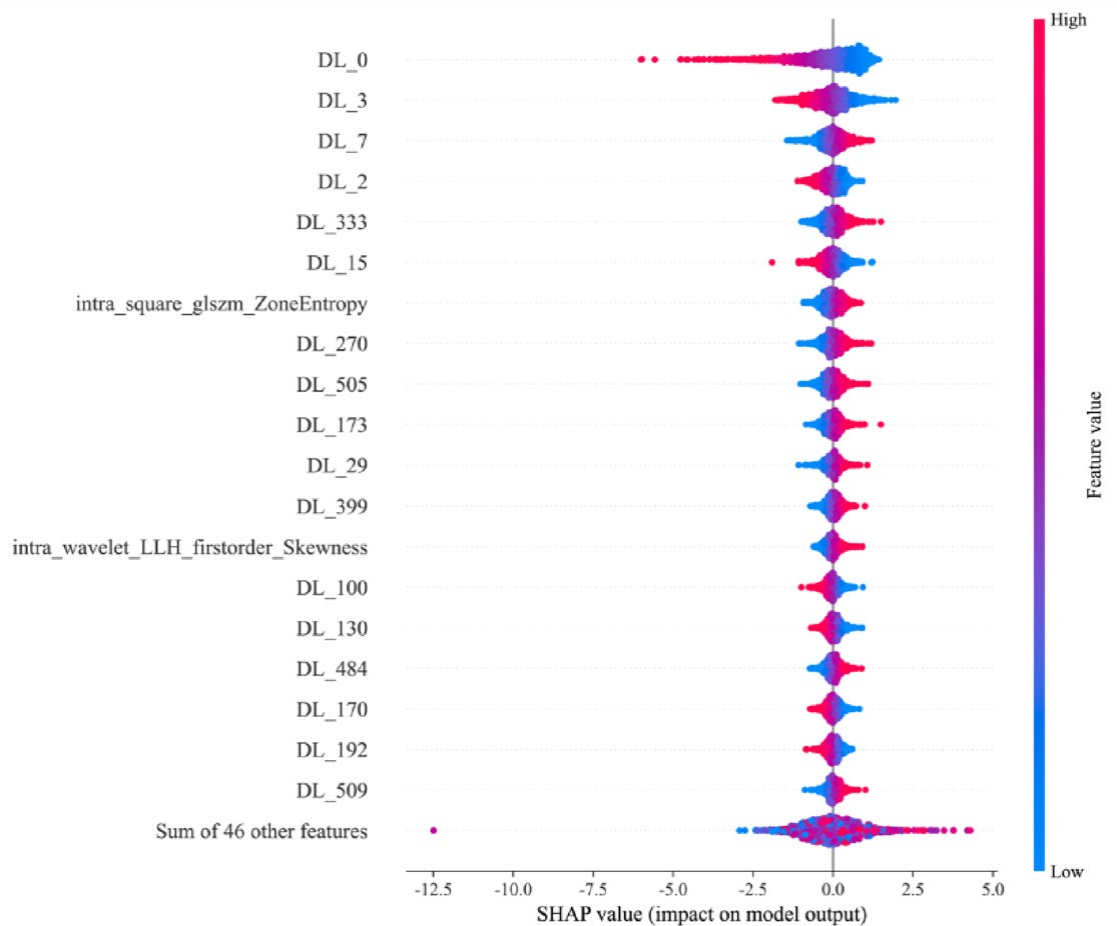
SHAP summary plots of the deep learning-radiomic model.

Model interpretability was evaluated using the SHAP framework and Grad-CAM. The SHAP summary plot (Fig. 4) used multidimensional visualization to show feature contributions in the logistic regression model. Features were ranked by importance on the vertical axis, with scatter density distributions showing SHAP value dispersion across the population. A blue-to-red color gradient indicated feature value magnitude, identifying nonlinear relationships between predictors and model outputs. Notably, the DL-0 radiomics feature showed strong discriminative power in distinguishing cases with or without gross ETE. This was seen in its left-skewed SHAP value distribution and the strong negative correlation between feature value decline and model output enhancement.

**Fig. 5:**
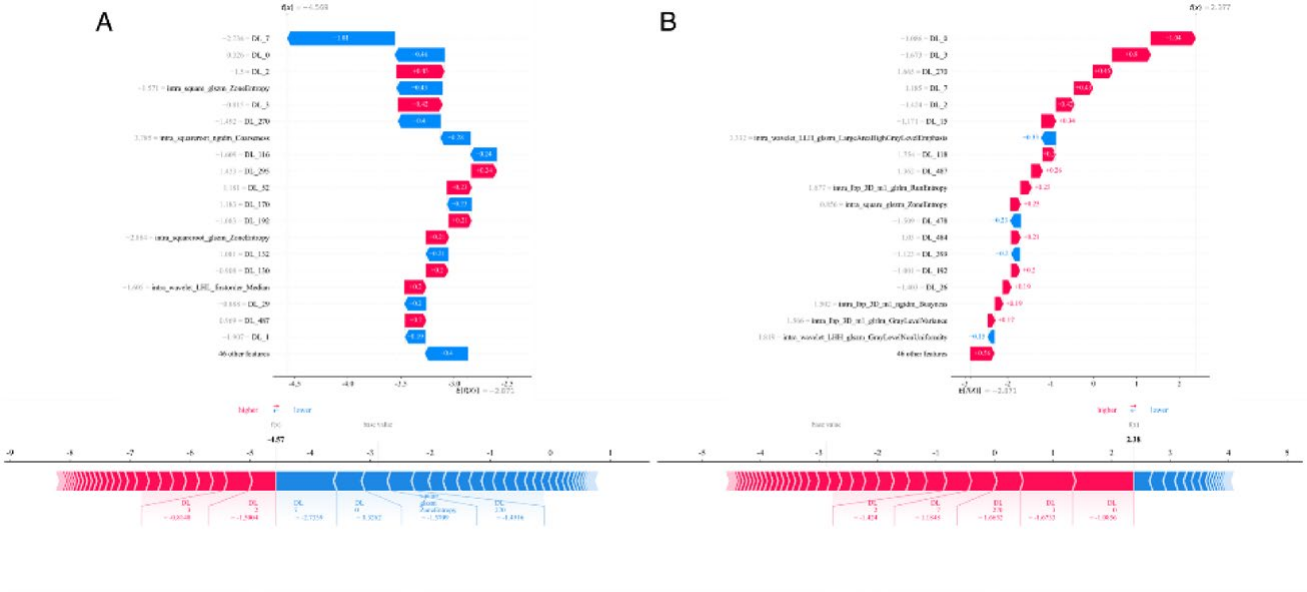
SHAP force plots.

For personalized predictions, SHAP force plots (Fig. 5) used a biomechanical vector visualization method. The baseline prediction (mean SHAP value of the cohort) served as the reference. The arrow length corresponded to the percentage contribution of each feature. Color coding (red for positive impact and blue for negative impact) clearly showed how each parameter influenced the final prediction probability. This dual visualization connected global feature importance with case-specific decision-making.

**Fig. 6:**
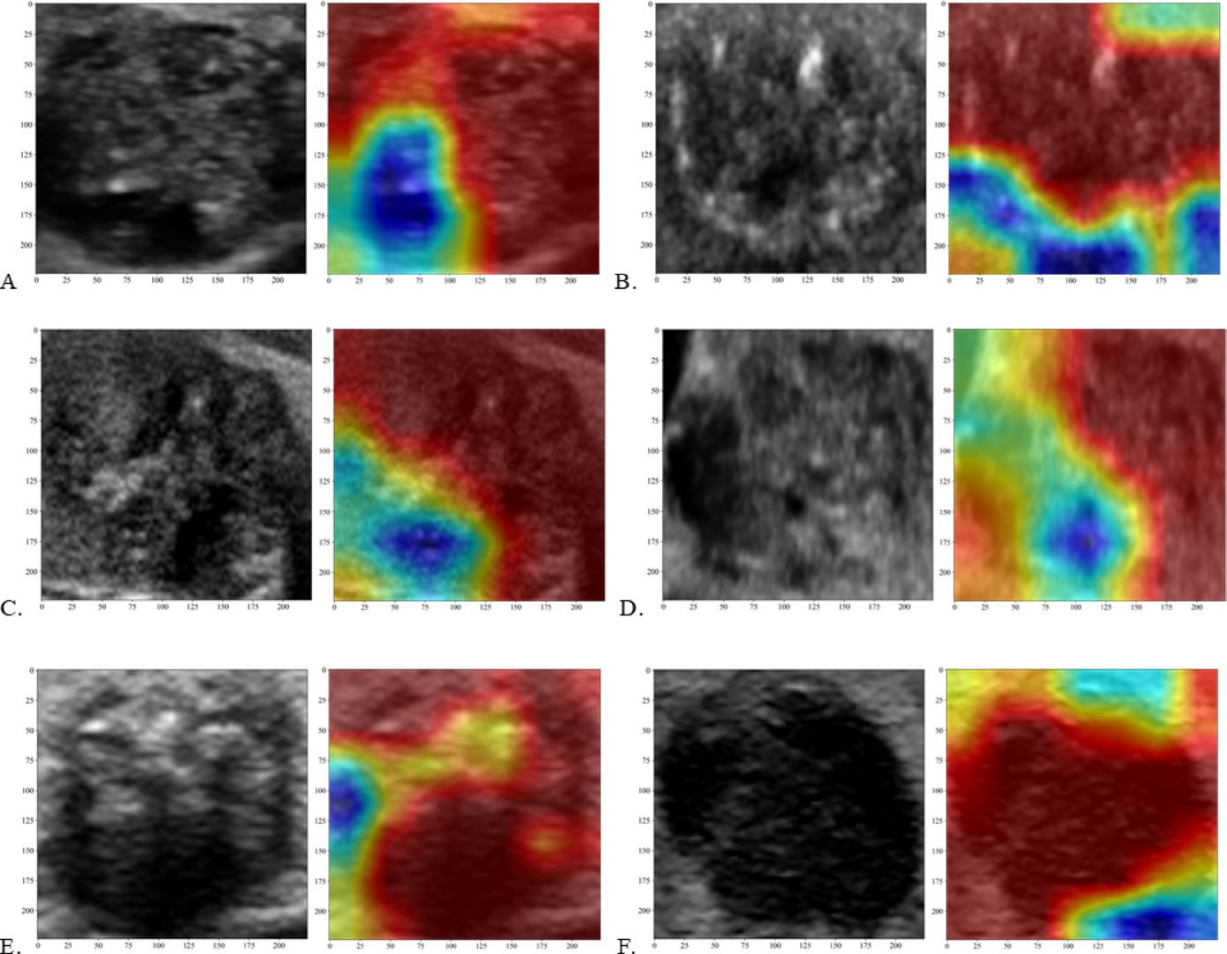
Heat maps for six US images (from six different PTC patients) displaying the importance of different image regions to the network decision of identifying the Gross ETE.

Grad-CAM technology was used to explain the spatial decision-making of deep learning models by analyzing gradient-flow correlations in the final convolutional layers. In nodules with gross ETE, activation regions (marked in blue) were mainly found at the tumor periphery transition zones and were relatively large. In nodules without extrathyroidal extension, feature responses (marked in red) were concentrated in the lesion cores (Fig. 6). This pixel level visualization helped decode key morphological decision factors, such as spiculated margins, abnormal aspect ratios, and intralesional calcification patterns, which were consistent with clinical diagnostic criteria.

The combined use of these techniques created a cognitive bridge between humans and AI. Grad-CAM heat maps visually showed radiologists the model’s focus areas, while SHAP analysis provided quantitative insights into feature-level decision-making. When there were differences between model predictions and clinical judgments, the spatial and quantitative evidence from these methods prompted productive interdisciplinary discussions. Importantly, this dual-modality interpretability framework increased clinical trust in AI systems by aligning computational decision-making with established oncological expertise.

## Discussion

In the present study, we developed a multicenter radiomics nomogram for preoperative identification of gross ETE in PTC patients, validated across diverse clinical settings. This individualized decision-support tool integrated multimodal predictors to optimize surgical planning precision.

Previous studies have proposed various models for predicting gross ETE. For example, Jiang et al.^13^ developed a radiomics nomogram using B-mode and contrast-enhanced ultrasound along with clinical features, achieving AUCs of 0.843 in training and 0.792 in validation. Similarly, Lu et al.^14^ established a preoperative model based on tumor location and size, with training and validation AUCs of 0.810 and 0.798, respectively. While these models demonstrated considerable diagnostic discrimination, they were limited by relatively small sample sizes and a lack of external validation. In contrast, our study developed a multimodal nomogram that integrated deep learning with radiomics and incorporated a broader set of clinical and ultrasound features. We specifically evaluated the diagnostic contributions of peritumoral regions by testing multiple expansion ranges (5, 10, 15, and 20 pixels). This has not only been confirmed by some previous high-quality studies, and has demonstrated excellent diagnostic efficacy in the model presented in this study^15–18^. The 15-pixel expansion generally yielded the best performance across most thresholds, although its advantage decreased at higher thresholds, suggesting that while peritumoral areas contained valuable information, excessive expansion may have introduced noise. Our final model, which used a 15-pixel expansion within an early fusion framework, achieved an AUC of 0.88 in predicting gross ETE in papillary thyroid carcinomas. This result surpassed prior studies, and also emphasized the clinical advantages of ultrasound as a noninvasive, cost-effective, and radiation-free imaging tool. Furthermore, our model was rigorously validated in a multicenter setting, reinforcing its robustness and potential for preoperative applications.

Our multi-modal feature fusion strategy significantly increased model performance. We innovatively identified intratumoral and peritumoral radiomics and deep learning features for gross ETE prediction, considering both regions and tumor spatial locations to comprehensively reflect tumor invasiveness. Recent studies indicate fusion models excel in medical image-based prediction tasks. For example, Gan et al. found feature fusion models enhanced the performance of radiomics and deep learning models in predicting KRAS mutations in rectal cancer on ERUS images by incorporating peritumoral regions^15^. Gu et al. showed that expanding tumor surrounding volume elements produced the best radiomics for predicting recurrence-free survival in pancreatic cancers^19^. The fusion model developed in this study demonstrated high specificity (0.888) in the test cohort, indicating its strong capability in eliminating non-ETE cases and potentially reducing unnecessary extensive surgeries. Although the sensitivity of the model remained moderate (0.630), meaning approximately 37% of true gross ETE cases could be missed, it still represented an improvement over previous reports. A previous study showed that the preoperative ultrasound detection of gross ETE by US physicians demonstrated a sensitivity of 30% and a specificity of 93%^4^.For example, Chung et al.^2^ reported a sensitivity of only 0.45 among radiologists in identifying the T3b-stage disease. When revisiting the model’s predictions of the model, we found that most correctly identified gross ETE cases exhibited apparent sternocleidomuscular invasions, suggesting that the model effectively recognized peritumoral signs such as capsular interruption or muscular involvement. In contrast, the majority of misclassified cases involved invasion of deeper structures such as the trachea, esophagus, or recurrent laryngeal nerve (RLN). The assessment of gross ETE involving the strap muscles or RLN poses a significant diagnostic challenge for imaging-based modalities, particularly ultrasound^20–22^. This can be partly attributed to the inherent limitations of ultrasound imaging, which does not always clearly visualize deep-seated nodules obscured by the trachea or esophagus. Moreover, the variable anatomical course of the RLN adds another layer of complexity. According to Newman et al.^23^, approximately 30% of nodules with RLN invasion are located away from the trachea, complicating diagnoses for both clinicians and AI models.

The clinical application of AI technology requires explainable models. Although radiomic features represent well-defined mathematical quantities, understanding how changes in their values impact predictions remains challenging. SHAP analysis addresses this problem by quantifying the contribution of each feature to the model prediction of gross ETE, revealing the positive or negative influence of different features, thereby enhancing model interpretability^24^. We also used the Grad-CAM method to highlight regions in the input image most critical for decision-making of the model (Fig. 6). This allowed both physicians and patients to intuitively understand where the “attention” of the model was focused, helping to mitigate the “black-box” nature of deep learning^25^. Furthermore, the final integrated model supported interpretability, because it was constructed using logistic regression and was effectively visualized using a nomogram. The combination of these interpretability methods should provide clinicians with greater confidence in using the model in clinical settings, because its predictions can be validated against medical expertise. This enhanced transparency should also potentially increase patients’ trust and acceptance of the rationale for using the model. Previous studies have also demonstrated the application of ResNet101 in assisting the diagnoses of thyroid disorders^26–28^. In the present study, we used transfer learning with a pretrained ResNet101 model and incorporated a large dataset. This approach effectively prevented overfitting in small sample data, and enhanced the model’s generalization ability, which is consistent with the original strategy of accelerating training and boosting model performance through transfer learning. Radiomics served as a more precise, objective, and efficient diagnostic approach, increasing the capabilities of conventional imaging diagnostics. By extracting hundreds of quantitative features from medical images and undergoing self-training and learning based on pathological outcomes, it provided valuable support for clinical decision-making^29,30^. In the radiomics model, texture features like aquare_glszm_ZoneEntropy significantly affected predictions, with higher values correlating with lower probabilities of extrathyroidal invasions. This likely indicated a relationship between tumor-margin texture roughness and tumor aggressive behavior. In the fusion model, deep learning features (e.g., DL_0) were the main contributors, showing superiority in capturing complex imaging features, as compared with traditional radiomics features.

This study had several limitations inherent to its retrospective design. First, although we incorporated multicenter data, inherent selection bias remained inevitable, necessitating future prospective validation with larger, multi-institutional cohorts. Second, while all ROI delineations underwent rigorous multi-reviewer verification, manual segmentation protocols may have still introduced measurement variabilities. Third, although preoperative ultrasound was our primary imaging modality, expanding data diversity through additional modalities (e.g., computed tomography and magnetic resonance imaging) could enhance model generalizability.

## Conclusion

In summary, our study showed that a deep learning and radiomics model based on ultrasound examination of the intratumoral and peritumoral regions was a noninvasive and effective way to identify whether PTC patients have gross ETE. In addition, we provided a SHAP value visualization model.

## Supplementary Information

Below is the link to the electronic supplementary material.

Supplementary Material 1

Supplementary Material 2

Supplementary Material 3

## Data Availability

Not applicable.

## References

[1] Amin, M. B. et al. The Eighth Edition AJCC Cancer Staging Manual: Continuing to build a bridge from a population-based to a more personalized approach to cancer staging. *CA Cancer J. Clin.* **67** (2), 93–99 (2017). 28094848 10.3322/caac.21388

[2] Tran, B. et al. An Analysis of The American Joint Committee on Cancer 8th Edition T Staging System for Papillary Thyroid Carcinoma. *J. Clin. Endocrinol. Metab.* **103** (6), 2199–2206 (2018). 29672723 10.1210/jc.2017-02551

[3] Chung, S. R. et al. Sonographic Assessment of the Extent of Extrathyroidal Extension in Thyroid Cancer. *Korean J. Radiol.* **21** (10), 1187–1195 (2020). 32729261 10.3348/kjr.2019.0983 PMC7458864

[4] Lamartina, L. et al. Can preoperative ultrasound predict extrathyroidal extension of differentiated thyroid cancer? *Eur. J. Endocrinol.* **185** (1), 13–22 (2021). 33886499 10.1530/EJE-21-0091

[5] Chen, S. et al. Machine learning-based pathomics signature could act as a novel prognostic marker for patients with clear cell renal cell carcinoma. *Br. J. Cancer*. **126** (5), 771–777 (2022). 34824449 10.1038/s41416-021-01640-2 PMC8888584

[6] Sato, M. et al. Machine-learning Approach for the Development of a Novel Predictive Model for the Diagnosis of Hepatocellular Carcinoma. *Sci. Rep.* **9** (1), 7704 (2019). 31147560 10.1038/s41598-019-44022-8 PMC6543030

[7] Shew, M., New, J. & Bur, A. M. Machine Learning to Predict Delays in Adjuvant Radiation following Surgery for Head and Neck Cancer. *Otolaryngol. Head Neck Surg.* **160** (6), 1058–1064 (2019). 30691352 10.1177/0194599818823200

[8] Ingwersen, E. W. et al. Dutch Pancreatic Cancer, Machine learning versus logistic regression for the prediction of complications after pancreatoduodenectomy. *Surgery* **174** (3), 435–440 (2023). 37150712 10.1016/j.surg.2023.03.012

[9] Patel, H., Shah, H., Patel, G. & Patel, A. Hematologic cancer diagnosis and classification using machine and deep learning: State-of-the-art techniques and emerging research directives. *Artif. Intell. Med.* **152**, 102883 (2024). 38657439 10.1016/j.artmed.2024.102883

[10] Hanahan, D. Hallmarks of Cancer: New Dimensions. *Cancer Discov*. **12** (1), 31–46 (2022). 35022204 10.1158/2159-8290.CD-21-1059

[11] Balachandran, V. P., Gonen, M., Smith, J. J. & DeMatteo, R. P. Nomograms in oncology: more than meets the eye. *Lancet Oncol.* **16** (4), e173–e180 (2015). 25846097 10.1016/S1470-2045(14)71116-7 PMC4465353

[12] Chen, L. C., Papandreou, G., Kokkinos, I., Murphy, K. & Yuille, A. L. DeepLab: Semantic Image Segmentation with Deep Convolutional Nets, Atrous Convolution, and Fully Connected CRFs. *IEEE Trans. Pattern Anal. Mach. Intell.* **40** (4), 834–848 (2018). 28463186 10.1109/TPAMI.2017.2699184

[13] Jiang, L. et al. Predicting Extrathyroidal Extension in Papillary Thyroid Carcinoma Using a Clinical-Radiomics Nomogram Based on B-Mode and Contrast-Enhanced Ultrasound. *Diagnostics (Basel)* **13**(10) (2023). 10.3390/diagnostics13101734 PMC10217699 37238217

[14] Lu, W. J. et al. Three-dimensional ultrasound-based radiomics nomogram for the prediction of extrathyroidal extension features in papillary thyroid cancer. *Front. Oncol.* **13**, 1046951 (2023). 37681026 10.3389/fonc.2023.1046951 PMC10482087

[15] Gan, Y. et al. Comparison of Intratumoral and Peritumoral Deep Learning, Radiomics, and Fusion Models for Predicting KRAS Gene Mutations in Rectal Cancer Based on Endorectal Ultrasound Imaging. *Ann. Surg. Oncol.* **32** (4), 3019–3030 (2025). 39690384 10.1245/s10434-024-16697-5

[16] Liu, K. et al. Improving the accuracy of prognosis for clinical stage I solid lung adenocarcinoma by radiomics models covering tumor per se and peritumoral changes on CT. *Eur. Radiol.* **32** (2), 1065–1077 (2022). 34453574 10.1007/s00330-021-08194-0

[17] Ding, J. et al. Optimizing the Peritumoral Region Size in Radiomics Analysis for Sentinel Lymph Node Status Prediction in Breast Cancer. *Acad. Radiol.* **29** (Suppl 1(Suppl 1), S223–S228 (2022). 33160860 10.1016/j.acra.2020.10.015 PMC9583077

[18] Shi, J. et al. MRI-based peritumoral radiomics analysis for preoperative prediction of lymph node metastasis in early-stage cervical cancer: A multi-center study. *Magn. Reson. Imaging*. **88**, 1–8 (2022). 34968703 10.1016/j.mri.2021.12.008

[19] Gu, Q. et al. Multiscale deep learning radiomics for predicting recurrence-free survival in pancreatic cancer: A multicenter study. *Radiother Oncol.* **205**, 110770 (2025). 39894259 10.1016/j.radonc.2025.110770

[20] Shindo, M. L. et al. Management of invasive well-differentiated thyroid cancer: an American Head and Neck Society consensus statement. AHNS consensus statement. *Head Neck*. **36** (10), 1379–1390 (2014). 24470171 10.1002/hed.23619

[21] Dralle, H. et al. Risk factors of paralysis and functional outcome after recurrent laryngeal nerve monitoring in thyroid surgery. *Surgery* **136** (6), 1310–1322 (2004). 15657592 10.1016/j.surg.2004.07.018

[22] Randolph, G. W. & Kamani, D. The importance of preoperative laryngoscopy in patients undergoing thyroidectomy: voice, vocal cord function, and the preoperative detection of invasive thyroid malignancy. *Surgery* **139** (3), 357–362 (2006). 16546500 10.1016/j.surg.2005.08.009

[23] Newman, S. K. et al. Invasion of a Recurrent Laryngeal Nerve from Small Well-Differentiated Papillary Thyroid Cancers: Patient Selection Implications for Active Surveillance. *Thyroid* **32** (2), 164–169 (2022). 34714169 10.1089/thy.2021.0310 PMC8861915

[24] Xiang, H. et al. Development and validation of an interpretable model integrating multimodal information for improving ovarian cancer diagnosis. *Nat. Commun.* **15** (1), 2681 (2024). 38538600 10.1038/s41467-024-46700-2 PMC10973484

[25] van der Velden, B. H. M., Kuijf, H. J., Gilhuijs, K. G. A. & Viergever, M. A. Explainable artificial intelligence (XAI) in deep learning-based medical image analysis. *Med. Image Anal.* **79**, 102470 (2022). 35576821 10.1016/j.media.2022.102470

[26] Lee, H., Kwak, J. Y. & Lee, E. Effective data selection via deep learning processes and corresponding learning strategies in ultrasound image classification. *Sci. Rep.* **15** (1), 16138 (2025). 40341123 10.1038/s41598-025-00416-5 PMC12062297

[27] Takano, Y. et al. Diagnosis of thyroid cartilage invasion by laryngeal and hypopharyngeal cancers based on CT with deep learning. *Eur. J. Radiol.* **189**, 112168 (2025). 40381388 10.1016/j.ejrad.2025.112168

[28] Agyekum, E. A. et al. Ultrasound-based classification of follicular thyroid Cancer using deep convolutional neural networks with transfer learning. *Sci. Rep.* **15** (1), 21708 (2025). 40592967 10.1038/s41598-025-05551-7 PMC12216321

[29] Sorrenti, S. et al. Artificial Intelligence for Thyroid Nodule Characterization: Where Are We Standing? Cancers (Basel) 14(14) (2022). 10.3390/cancers14143357 PMC9315681 35884418

[30] Park, V. Y. et al. Combining radiomics with ultrasound-based risk stratification systems for thyroid nodules: an approach for improving performance. *Eur. Radiol.* **31** (4), 2405–2413 (2021). 33034748 10.1007/s00330-020-07365-9

