# Supplementary material for: Innovative fusion models: elevating preoperative gross ETE prediction in thyroid cancer patients
Source: Sci Rep. 2026 Mar 11;16:13070. doi: 10.1038/s41598-026-43230-3 (PMC13099980; doi:10.1038/s41598-026-43230-3)
Supplement: Supplementary file 3 — Supplementary Material 3 [file 41598_2026_43230_MOESM3_ESM.docx]

# Additional file

# Supplementary Material

## Table of Contents

## Methods

Method S1. Sample size estimation

Method S2. Equipment and instruments

Method S3. Training process of platform

Method S4. Detailed training process of our DLR model

## Figures

Figure S1. Flowchart of patient enrollment and allocation.

Figure S2.Cross-validation plots, coefficient plots and Feature weight histogram for feature selection utilizing the LASSO algorithm

Figure S3. Sample prediction histogram

Figure S4. Confusion matrices for the comprehensive diagnoses from the RAD model, DL model and the DLR model on train and validation cohorts

Figure S5.Delong , NRI and IDI test between three models

## Tables

Table. S1. Machine learning of Radiomics and Deep learning.

Table. S2. Machine learning of DLR model.

Method S1. Sample size estimation

The sensitivity (P) and specificity (P) of the predictive model in discovery dataset were 0.784 and 0.692, respectively. We set Zα0.05=1.96 and allowable error (δ)=0.2P, sample size of case and control was calculated according to the above formula of sample size calculation in diagnostic experiment, respectively;The sample size was calculated according to the following sample size calculation formula:


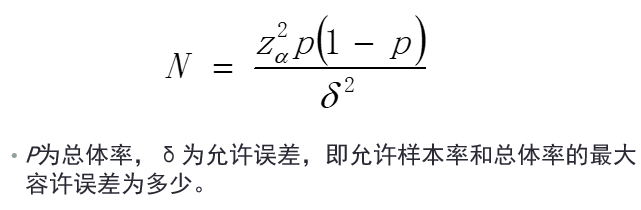


N =200 cases were calculated, that is, 100 samples are required for each diagnostic test. Meanwhile, the two diagnostic methods examine the same sample separately. Considering 15% loss to follow-up and rejection, at least 118 cases are included, which indicates that the current sample size of 300 cases in the datasets are enough.

Method S2. Equipment and instruments

Color Doppler ultrasound machines equipped with a real-time, high-frequency (5-10 MHz) linear-array probes are used in this study.

Method S3. Training process of platform

The training objective of our radiomics model is based on the AI onekey platform. Our method was implemented in Pytorch 1.0 and was performed on a personal computer with an Intel Core i9-13900k CPU, 128 GB memory, and one GeForce RTX 4090 GPU for computing acceleration.


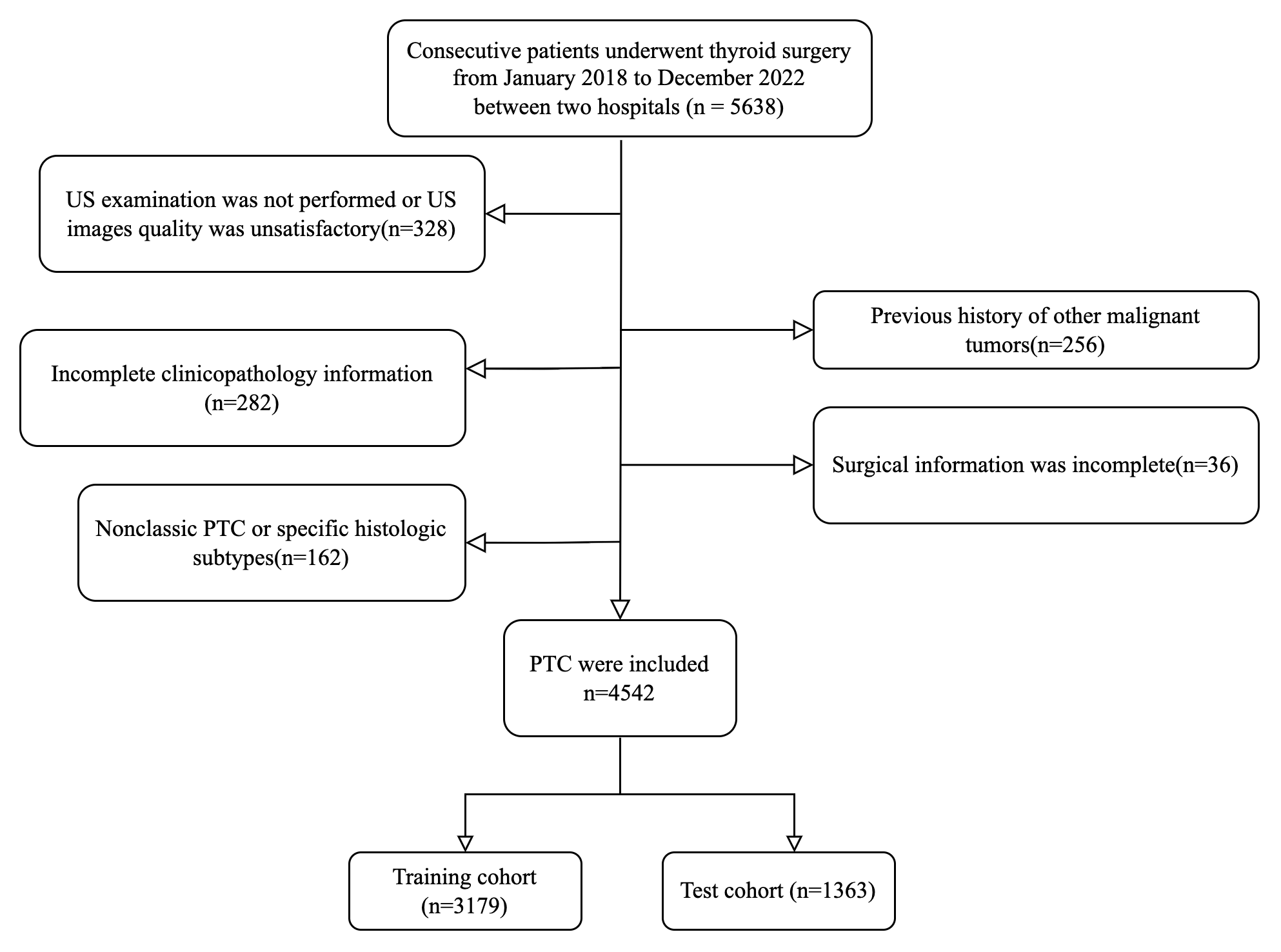


**Figure S1. Flowchart of patient enrollment and allocation.**


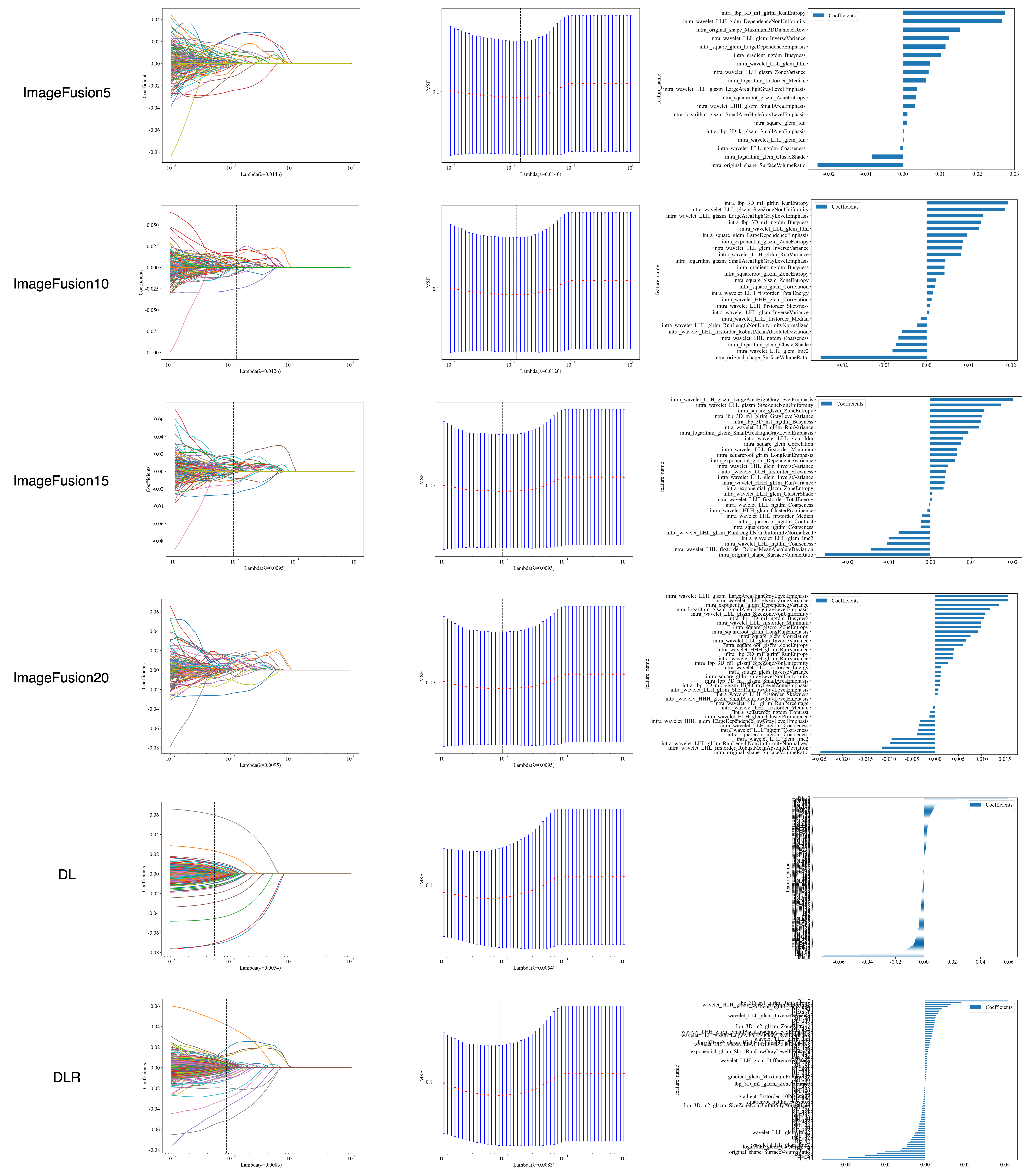


**Figure. S2 Cross-validation plots, coefficient plots and Feature weight histogram for feature selection utilizing the LASSO algorithm:**


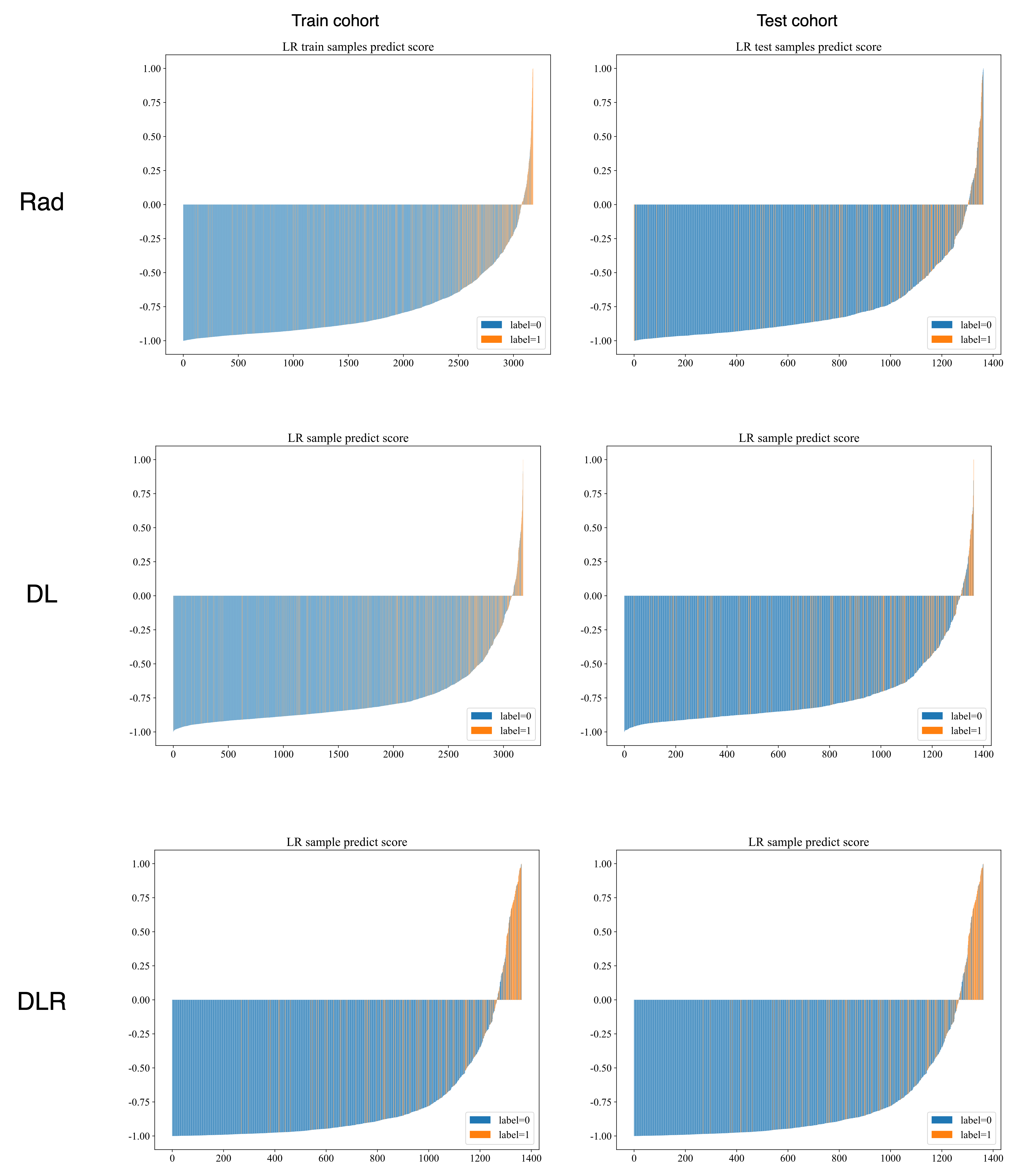


**Figure S3. Sample prediction histogram**


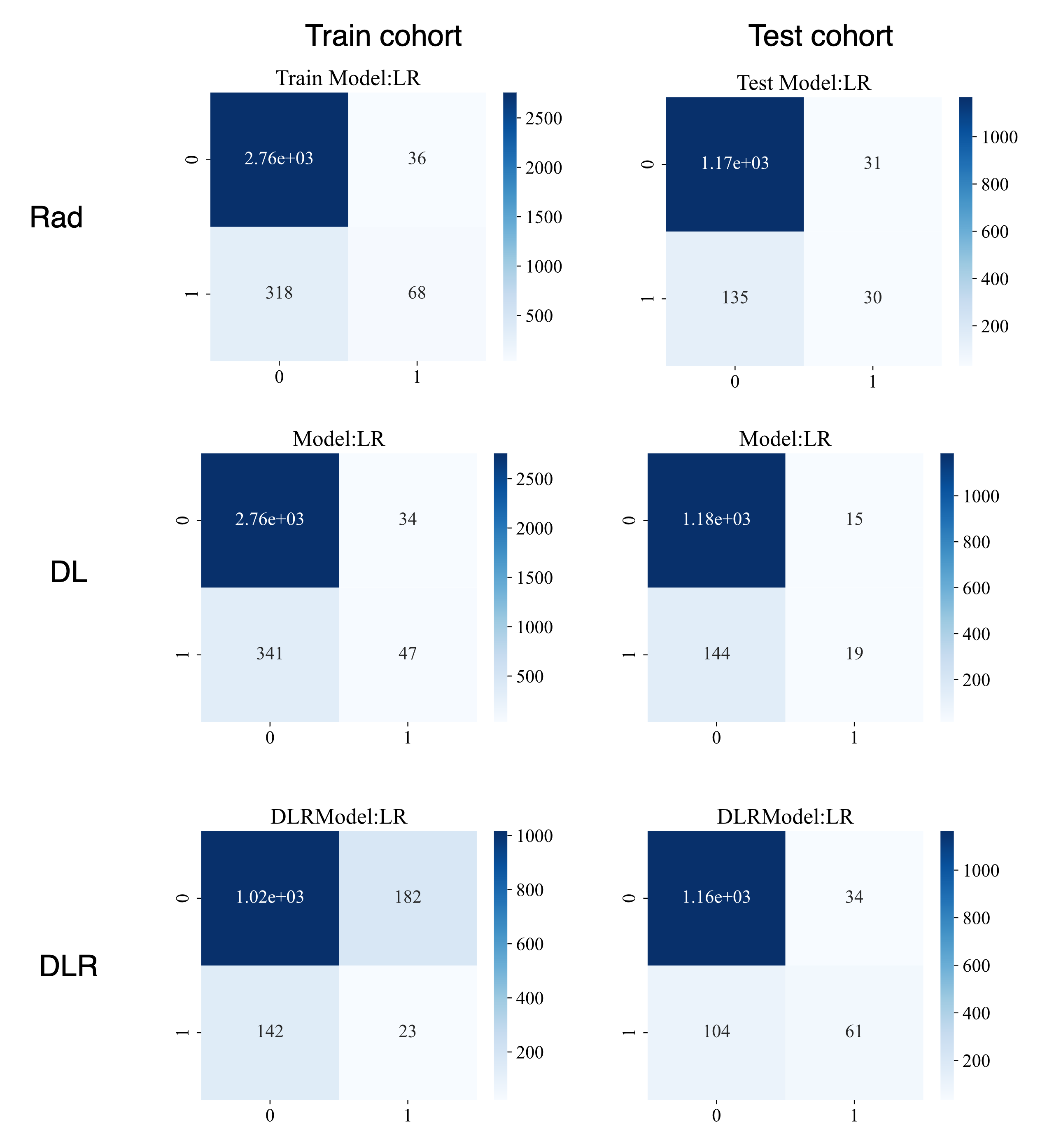


**Fig S4. Confusion matrices for the comprehensive diagnoses from the Intra model and the Imagefusion model on train and test cohorts**


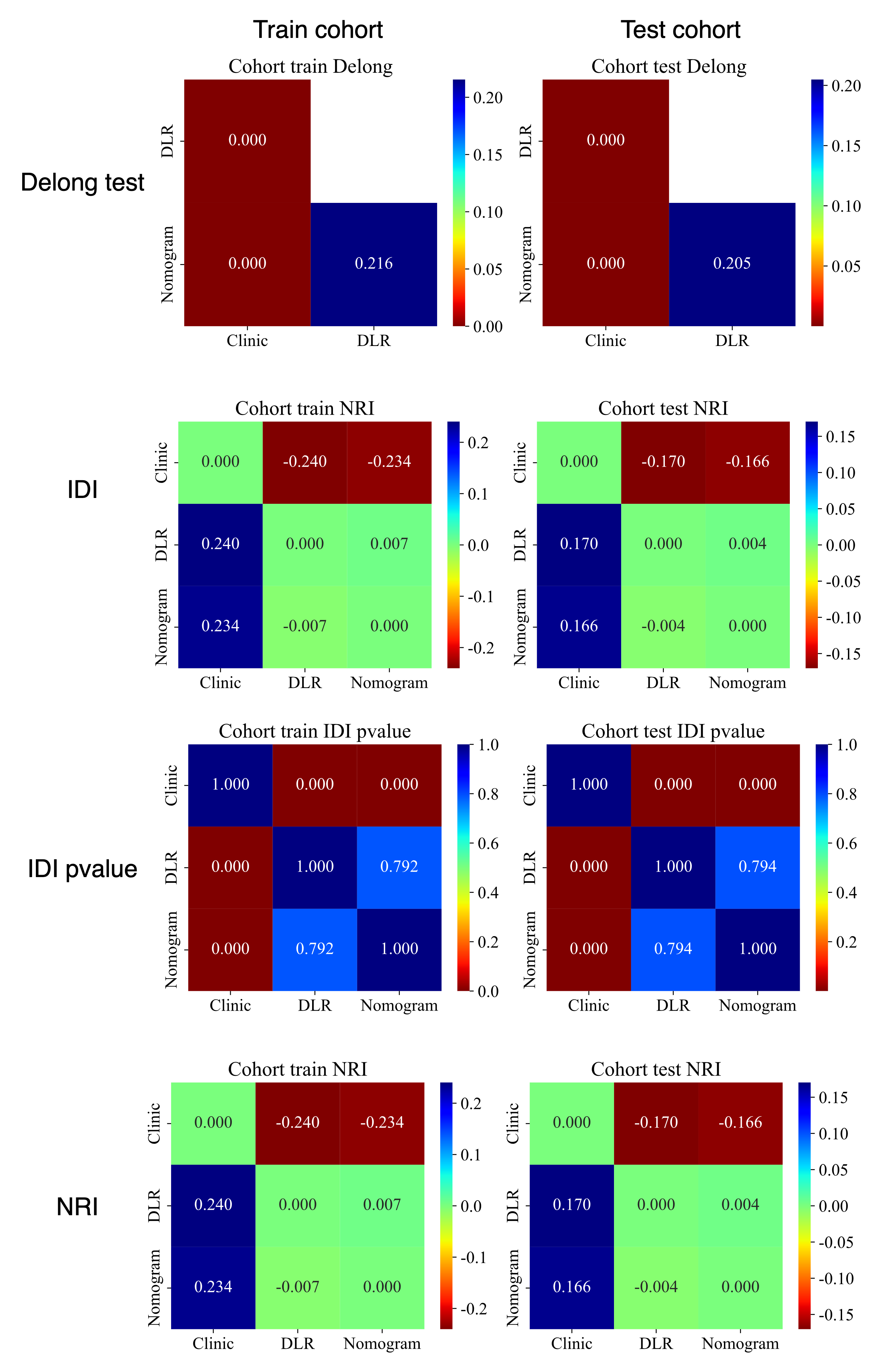


**Figure S5. Delong , NRI and IDI test between different machine learning.**

DeLong's Test is a nonparametric statistical method primarily used to compare the differences in the Area Under the ROC Curve (AUC) between two predictive models. By constructing covariance matrices, this test evaluates whether the observed differences in diagnostic or predictive performance between models (e.g., medical diagnostics or machine learning models) are statistically significant. Unlike traditional parametric tests, DeLong's Test does not require assumptions about data distribution, making it more versatile for real-world applications.

The NRI metric evaluates improvements in classification accuracy by measuring the net proportion of correct reclassifications. It assesses two dimensions:Event group: The proportion of correctly "upgraded" cases (reclassified from low-risk to high-risk).Non-event group: The proportion of correctly "downgraded" cases (reclassified from high-risk to low-risk).The NRI is calculated as the sum of these two proportions, providing a comprehensive measure of a model's ability to refine risk stratification.

The IDI metric systematically quantifies the overall improvement in predicted probabilities when comparing a new model to a reference model. It calculates the weighted average of prediction probability differences across two groups: the event group (where outcomes occur) and the non-event group (where outcomes do not occur). Specifically, IDI represents the improvement in the average predicted probability for the event group minus the reduction in the average predicted probability for the non-event group. A positive IDI value indicates enhanced discriminative power in distinguishing high-risk from low-risk individuals.
